# Early plant growth and bacterial community in rhizoplane of wheat and flax exposed to silver and titanium dioxide nanoparticles

**DOI:** 10.1007/s11356-018-3346-7

**Published:** 2018-10-04

**Authors:** Anna Gorczyca, Sebastian W. Przemieniecki, Tomasz Kurowski, Magdalena Oćwieja

**Affiliations:** 1 Department of Agricultural Environment Protection, University of Agriculture in Krakow, Mickiewicza 21, 31-120 Krakow, Poland; 2 Department of Entomology, Phytopathology and Molecular Diagnostics, University of Warmia and Mazury, Prawochenskiego 17, PL-10-721 Olsztyn, Poland; 3 Jerzy Haber Institute of Catalysis and Surface Chemistry, Polish Academy of Sciences, Niezapominajek 8, 30-239 Krakow, Poland

**Keywords:** Silver nanoparticles, Titanium dioxide nanoparticles, *Triticum aestivum*, *Linum usitatissimum*

## Abstract

Silver and titanium dioxide nanoparticles (AgNPs and TiO_2_NPs) are highly useful, but they are also a significant reason for concern as they exert toxicity. The goal of research was to assess the role of three kinds of NPs in concentrations of 100 mg L^−1^ on early growth plants (wheat, flax) and bacterial community in rhizoplane. Titanium (IV) oxide anatase (TiO_2_NPs1) and titanium (IV) oxide nanopowder (TiO_2_NPs2) are commercial products. A suspension of AgNPs was prepared via a procedure of reduction with tannic acid. The response of Monocot and Dicot growth form plants to the tested NPs was different. Germination and seedling growth of wheat treated with TiO_2_NPs1 was better. The response of flax to NPs was noted as an increase of chlorophyll content. The bacterial community in wheat rhizoplane was not significantly modified, but there was a declining trend. In turn, a difference in the surface charge of NPs had an influence on the total bacterial community in Dicot rhizoplane. Positively charged TiO_2_NPs2 significantly decreased the quantity of total bacteria in contrast to negatively charged AgNPs and TiO_2_NPs1 which increased it. A qualitative analysis did not confirm the influence of the surface charge of NPs on an increase/decrease in the quantity of *Pseudomonas* and *Bacillus* bacteria, but did show that there was no toxicity of the tested NPs to the plant growth-promoting bacteria community. The rhizoplane microbiome was dependent on the species of plant, and the bacteria found in the communities are sensitive to NPs to a varying degree.

## Introduction

Many studies related to nanoparticles (NPs) show that their release has a negative influence on ecosystems and human health (Nel et al. 2013; Cox et al. 2016). Despite the fears, knowledge on this subject is still insufficient. In the case of plant growth and development, NPs are noted to have a positive and a negative influence (Montes et al. 2017). Rhizoplane microbiome is an important factor in the development of plants but for their part, microorganisms, being particularly sensitive to changes in the environment, in most cases respond negatively to NPs (Hegde et al. 2016). NPs of silver (Beddow et al. 2014) and of titanium oxide (Ge et al. 2011) have been found to be toxic to bacteria in vitro. Other effects may occur in the soil, where physicochemical properties, texture, and organic matter, may change the properties of NPs. Such an interaction may result in increased or decreased bioavailability and toxicity of NPs for plants and microorganisms (Dimkpa 2014). Many studies confirm that exposure to NPs does not change the structure and function of soil microbial communities, while others determine the impact to be negative (Simonin and Richaume 2015). Microorganisms in the rhizosphere influence plants in a number of ways: they activate growth, nutrition, development, susceptibility to diseases, resistance to heavy metals, and degradation of xenobiotics. The microbiota occurring in the roots of plants above all mobilizes and provides nutrients by increasing their bioavailability. A non-nutritional function is greater resistance to biotic and probably abiotic stresses too (Bulgarelli et al. 2013). The results of the interaction of NPs with biological systems vary, depending on the experimental environment. The influence of NPs in combined plant-microbial systems has been assessed in a small number of studies, compared to the relatively large number of separate assessments pertaining to plants and microorganisms (Dimkpa 2014).

For this reason, the aim of the research was to preliminary assess a plant**-**rhizoplane microbiome system, which was subjected to selected NPs, found to be particularly useful in many technological applications, i.e.**,** silver nanoparticles (AgNPs) and titanium oxide nanoparticles (TiO_2_NPs). These nanoparticles have in numerous cases been shown to have a negative effect in vitro. The studies intentionally differentiated plants species to Monocot (wheat) and Dicot (flax) growth form using one concentration of NP. Differences in the development of root system and cell wall chemistry between Monocot and Dicot may affect rhizoplane microbiome. This test was carried out in soil conditions. The intentions of evaluating initial growth (germination, seedlings biometry, relative chlorophyll content) and basic parameters of the rhizoplane microbiome (total bacteria, *Pseudomonas*, *Bacillus*, *Clostridium*, toxinogenic *Penicillium*) was to select the hot spot of the NPs activity in systems. This will allow focusing detailed, systematic studies on the impact of NPs on the biological system of the plant**-**rhizoplane microbiome in soil conditions.

## Materials and methods

All chemical reagents used in the experiments (silver nitrate, tannic acid, ammonia solution 25%) are commercial products available from Sigma Aldrich (Switzerland). Similarly, the titanium (IV) oxide anatase (TiO_2_NPs1) and titanium (IV) oxide nanopowder (TiO_2_NPs2) were purchased from Sigma Aldrich. These compounds were used without further purification. Aqueous solutions of these compounds were prepared with the use of ultrapure water (conductivity of 0.06 μS·cm^−1^) obtained from a Milli-Q Elix & Simplicity 185 purification system (Millipore SA Molsheim, France).

A suspension of silver NPs was prepared according to the procedure described in our previous work (Barbasz et al. 2015). Briefly, the silver nitrate aqueous solution (320 mL, 1 mM) was mixed with the tannic acid solution (10 mL, 0.6 mM) and then the pH of the mixture was adjusted using ammonia solution to the value of 9. After 1 hour of mixing at room temperature, the silver nanoparticle suspension obtained was purified using an ultra-filtration method. The mass concentration of silver in the suspensions was determined using a DMA5000M density meter (Anton Paar, USA), as described previously (Barbasz et al. 2015). In the experiments with the plant culture, the suspensions of TiO_2_NPs and of AgNPs were applied in concentrations of 100 mg·L^−1^. Similarly, the silver nitrate solution with a concentration of silver ions equal to 100 mg·L^−1^ was applied. The electrical conductivity and pH of the colloidal suspension were measured using CPC-505 multifunctional device (Elmetron, Poland) equipped with an EC-60 conductivity sensor and an EPS-1 glass electrode (Elmetron, Poland). The morphology and size distribution of the applied NPs were determined by means of a microscope examination method using a JEOL JSM-7500F microscope (USA). The silver NPs were visualized in transmission mode (TEM). For this purpose, a drop of the silver suspension was placed on a copper grid coated with carbon film. The solvent was evaporated at an elevated temperature (50 °C) and the samples were visualized. In the case of the titanium dioxides, the powders supplied were glued to the special conductive holder using carbon tape and were imaged in the secondary electron mode (SEM). The micrographs were analyzed using MultiScan software supplied by Computer Scanning System. The size of the silver NPs and of both types of the titanium dioxide NPs, in the suspension of a concentration of 100 mg·L^−1^, was determined by the dynamic light scattering (DLS) technique via diffusion coefficient measurements carried out using a Zetasizer Nano ZS instrument (Malvern, UK). The hydrodynamic diameter of the NPs was calculated based on the Stokes–Einstein relationship (Barbasz et al. 2015). The electrokinetic properties of the NPs under controlled conditions of pH, ionic strength, and temperature were determined via electrophoretic mobility measurements carried out using the Zetasizer Nano ZS instrument. The zeta potential of the NPs was calculated using Henry’s law (Barbasz et al. 2015). The concentration of silver ions released from the NPs after 14, 30, and 60 days was determined using Seven Compact™ pH/ionometer (Mettler Toledo, Poland) equipped with a perfectION™ silver/sulfide electrode. The dissolved oxygen (DO) concentration in the suspensions of controlled pH, ionic strength, and the silver NPs concentration was measured using a COG-1 t oxygen probe connected with a CPO-505 oxygen meter (Elmetron, Poland). In order to separate the NPs from silver ions, the ultrafiltration method described in our previous work (Barbasz et al. 2017) was applied.

The plants used in the experiments were as follows: common spring wheat cv. Bombona and common flax cv. Nike. Sixty seeds of tested plants were put in plastic pots filled with 0.8 kg of homogeneous soil with about 40% maximum water capacity. Next, the soil was evenly soaked with solution (1 mL in 1 L sterile water). Control objects were soaked with sterile water only. Seedlings were allowed to grow up in a phytotron under controlled conditions at 20 °C day and 18 °C night temperature, a 12-h photoperiod with a light intensity of 220 μmol photons m^−2^·s^−1^ PPFD, and 80% RH. The soil was supplemented with water every 3 days in the same amount for each treatment. First, 5 days of growth was allowed to proceed with foil cover and then the germination of seeds was controlled. Germination percentage was measured using the following formula: germination [%] = (number of seed germinated/total number of seeds) × 100. After 7 days, the plants were sprayed with the same solutions that were applied the first time to the soil. After 21 days, the plants were analyzed to determine their biometric and relative chlorophyll content (Chlorophyll Meter SPAD-502 Plus Konica Minolta INC, Japan). Total rhizoplane bacteria, *Clostridium* spp. and toxin-producing *Penicillium* were amplified with the Maxima Probe qPCR Master Mix (2X) (Thermo Fisher Scientific, USA) according to the methods used by Yu et al. (2005), Song et al. (2004), and Suanthie et al. (2009), respectively. The phlD gene of PGP (plant growth promoting) *Pseudomonadaceae* was determined according to the method proposed by Hu et al. (2016). To determine *Bacillus* spp. 16SBACF and 16SBACR primers (Mora et al. 2011) were used. Both *Pseudomonadaceae* and *Bacillus* amplified with the Maxima Sybr Green qPCR Master Mix (2X) (Thermo Fisher Scientific, USA). Efficiency of reaction was 0.91 to 1.00. The resulting data was analyzed using *STATISTICA* software. Duncan’s multiple range test (*p* < 0.01) was used to compare the means. The principal component analysis (PCA) was performed by Pearson (correlation) method. Agglomerating hierarchical clustering was performed by Bray and Curtis dissimilarity statistics with Ward’s agglomeration method. Both calculations were made with XLSTAT program (Addinsoft, UK).

## Results and discussion

Selected properties of the colloidal suspensions and the NPs were determined and it is shown in Table 1. The response of the plant-rhizoplane microbiome system to NPs is presented in Tables 2 and 3. The treatments applied had a limited influence on the germination and early development of the shoots of wheat and flax seedlings compared to the control. This response was different for the two plants tested (Monocot and Dicot growth forms). Enhanced seed germination and seedling growth was only obtained in wheat treated with TiO_2_NPs1. Feizi et al. (2012) reported significantly faster germination of wheat in response to treatment with TiO_2_NPs and higher shoot and seedling lengths than with untreated wheat, but only at low concentrations, i.e., of 1–2 mg·L^−1^ and no significant effect at concentration of 100 mg·L^−1^. In our experiment, the response of the Dicot growth form, i.e., flax, was weaker and only an enhanced trend was observed. Often, TiO_2_NPs have been noted to increase the germination rate and growth of some plants (Zhang and Karan 2005; Lyu et al. 2017) but under laboratory, in vitro conditions. Positive effects of TiO_2_NPs on germination and early growth of plants cultivated in soil conditions were found in our experiment but only in some cases. The NPs applied did not modify the dry mass of either of the plants in our experiment. Differences were noted in the greenness of leaves which indicates changes in the chlorophyll content of the treated plants. AgNO_3_ decreased the chlorophyll content of both plants treated. The response of flax to NPs was noted as an increase in chlorophyll content. The total bacterial community in wheat rhizoplane was not significantly modified, but there was a declining trend. In turn, there was a modification in the total quantity of bacteria in the flax rhizoplane in the case of the treatments with NPs. The difference in surface charge determined in the characteristics of the compounds used had a decisive influence on the bacterial community in the flax rhizoplane. Positively charged TiO_2_NPs2 significant decreased the quantity of bacteria in contrast to the negatively charged AgNPs and TiO_2_NPs1 which increased it. Abbaszadegan et al. (2015) have proven that the surface charge of AgNPs is a significant factor affecting bactericidal activity-positively charged NPs showed a high level of effectiveness against five species of pathogenic bacteria. Those authors explain the response based on van der Wal et al. (1997), citing the fact that the cellular membrane of the bacteria has a negative charge due to the presence of carboxyl, phosphate, and amino groups. The repulsion between the bacteria and negatively charged NPs could result in the formation of an electrostatic barrier by limiting the interaction between the NPs and the bacteria. The same conclusion is presented by Zhu et al. (2012). The cell membrane consists of an anionic hydrophilic outer surface. In contrast to neutral or anionic NPs, cationic particles attach more readily to the cell surface, from where they may also be taken up more avidly if size permits.

**Table 1:** Selected physicochemical properties of NPs suspension

Properties	Type of colloid		
	AgNPs	TiO_2_NPs1	TiO_2_NPs2
Concentration [mg L^−1^]	100	100	100
Conductivity [μS cm^−1^]	9.4	5.4	8.6
pH	5.9	5.4	5.5
Particle size [nm]*	16 ± 4	207 ± 17	68 ± 7
Diffusion coefficient [cm^2^ s^−1^]**	3.1·10^−7^	1.1·10^−8^	6.1·10^−9^
Hydrodynamic diameter [nm]**	17 ± 3	441 ± 40	812 ± 30
Electrophoretic mobility [(μmcm) (Vs)^−1^]**	− 3.41 ± 0.09	− 3.74 ± 0.05	+ 2.13 ± 0.04
Zeta potential [mV]**	− 63 ± 2	− 58 ± 1	31 ± 1
Concentration of leached silver ions from suspensions**DO = 7.4 mg L^−1^ after 14/30/60 days	7 ± 1.4/11 ± 2.2/10 ± 3.2	–	–

*DO* dissolved oxygen

*Determined from size distribution derived from TEM micrographs; **for 100 mg L^−1^, and *T* = 298 K

**Table 2:** Effect of NPs on tested parameters of plants seedlings

Plant	Treatment	Germination [%]		Leafs weight [g]		Leafs length [cm]		Dry matter [%]		Greennessof leaves [SPAD]	
Common wheat	control	68	b*	4.80	b	28.21	a	12.8	a	34.04	ab
	AgNO_3_	64	b	4.91	ab	27.10	a	14.2	a	30.34	c
	AgNPs	72	b	5.19	ab	27.32	a	14.9	a	32.35	b
	TiO_2_NPs1	84	a	5.40	a	29.51	a	14.3	a	33.63	ab
	TiO_2_NPs2	64	b	5.19	ab	30.25	a	13.3	a	34.42	a
	Mean	70		5.10		28.21		13.9		32.96	
Common flax	control	75	ab	2.66	b	9.65	a	8.05	a	21.93	b
	AgNO_3_	63	b	0.78	c	5.62	b	8.23	a	15.01	c
	AgNPs	77	a	3.25	a	9.72	a	6.85	a	26.23	a
	TiO_2_NPs1	78	a	2.86	b	9.63	a	7.85	a	25.25	ab
	TiO_2_NPs2	80	a	2.90	b	9.31	a	6.85	a	26.23	a
	Mean	75		2.49		8.78		6.58		19.72	

*Values marked with the same letters in columns are not significantly different (*p* ≤ 0.05) according to the multiple Duncan’s test

**Table 3:** Changes on load of selected group of bacteria in root zone after NPs treatment [μg·g^−1^ DM]

Plant	Treatment	Bacterial DNA		PGP *Pseudomonas*		*Bacillus* spp.		*Clostridium* spp.		Toxinogenic *Penicillium* spp.	
Common wheat	control	6.17	a*	2.50	ab	2.6·10^−4^	b	0.22	a	52.2·10^−4^	c
	AgNO_3_	5.12	a	0.19	b	1.3·10^−4^	b	0.12	b	0	a
	AgNPs	5.49	a	1.41	ab	1.4·10^−4^	b	0.13	b	8.6·10^−4^	b
	TiO_2_NPs1	5.63	a	2.91	a	1.9·10^−4^	b	0.16	ab	0	a
	TiO_2_NPs2	5.37	a	2.99	a	3.9·10^−4^	a	0.17	ab	0.1·10^−4^	a
	Mean	5.56		2.00		2.2·10^−4^		0.16		12.2·10^−4^	
Common flax	control	10.15	c	0.08	b	1.5·10^−5^	c	0.17	a	ND	
	AgNO_3_	7.48	e	0.09	b	2.6·10^−5^	bc	0.06	b	ND	
	AgNPs	13.32	a	0.54	a	3.9·10^−5^	ab	0.07	b	ND	
	TiO_2_NPs1	11.43	b	0.09	b	2.6·10^−5^	bc	0.14	a	ND	
	TiO_2_NPs2	9.23	d	0.20	b	5.7·10^−5^	a	0.05	b	ND	
	Mean	10.32		0.20		2.7·10^−5^		0.09		–	

*DM* dry matter, *PGP* plant growth promoting, *ND* no detected (obtained results were below the lowest calibration curve values)

*Values marked with the same letters in columns are not significantly different (*p* ≤ 0.05) according to the multiple Duncan’s test

Further analysis of the bacterial community conducted in our own study for rhizoplanes of wheat and flax showed specific quantitative modifications under the influence of the applied NPs, most often depending on the plant species. The *Pseudomonas* community rich in wheat rhizoplane was stimulated by TiO_2_NPs independently of the surface charge and did not differ from the control under treatment with AgNPs. In turn, significant growth was observed for the community of *Pseudomonas* that was poor in flax rhizoplane under treatment with AgNPs. There was significant growth in the quantity of *Bacillus* spp. in the wheat rhizoplane only under treatment with TiO_2_NPs2, and in the flax rhizoplane, such growth was caused by all NPs used in the study, whereby TiO_2_NPs2 were found to have the most significant effect. *Clostridium* spp. was significantly limited in the rhizoplane of both species of plant by AgNPs, and to a lesser degree by TiO_2_NPs. The study showed toxinogenic *Penicillium* bacteria in the wheat rhizoplane, and all the treatments used reduced it in quantity. The qualitative study conducted did not confirm the surface charge of NPs to have any influence on an increase/decrease in the quantity of bacteria in the rhizoplanes of both species of plant. However it appears to be significant that the tested NPs were not shown to have any toxic effects on the community of plant growth-promoting bacteria. It was also confirmed that the rhizoplane microbiome is dependent on the species of plant (Lemanceau et al. 1995), and that the bacteria found in the communities are sensitive to NPs to a varying degree.

Evidence of the toxic effect of some AgNPs on bacteria in a soil system has been provided (Anjum et al. 2013; Tripathi et al. 2017). Exposure of soil bacteria to 100–1000 mg L^−1^ of various types of AgNPs has been shown to significantly inhibit function. Calder et al. (2012) evaluated *Pseudomonas bacteria* in sand and soil matrices and found evidence of toxicity of AgNPs at a very low concentration in sand only. Organic matter and the changes in surface charge of NPs involved eliminated their toxicity. Our results showed no toxic effects of negatively charged tannin-reduced AgNPs on both the plants tested and rhizoplane bacteria. A toxic effect was observed for the ionic form of Ag and flax was much more sensitive to this. Silver ions had a significant negative effect on the germination and growth of flax seedlings and significantly increased the quantity of bacteria in flax rhizoplane.

The results obtained for treatment with TiO2NPs and ionic silver (AgNO3) are consistent with the analysis provided by Cox et al. (2016). Studies analyzed according to Cox et al. (2016) have shown that the toxicity of AgNPs is dependent on the properties of the form that results from the method of reduction applied. Most of the silver NPs examined exhibit phytotoxicity and bacteriostaticity at fairly low concentrations. However, some types of AgNP coating resulting from the reduction method applied have a significantly reduced toxic effect on living organisms, which is important for the protection of the environment. Yang and Watts (2005) indicated that surface area and surface characteristics play an important role in the phytotoxicity of AuNPs too.

Razzaq et al. (2016), by using soil-applied AgNPs synthesized by a reduction of AgNO_3_ with trisodium citrate at a concentration 100 mg L^−1^ shows higher weights of wheat seedlings and higher chlorophyll content too. But the same NPs caused significant reduction in wheat germination in comparison to the control under in vitro conditions. The effects of AgNPs on the inhibition of germination have been reported for many plants in not-soil conditions (Barrena et al. 2009; El-Temash and Joner 2012). This shows that there is another decisive factor in addition to the surface properties of NPs, i.e., the conditions under which plants are treated.

**Fig. 1:**
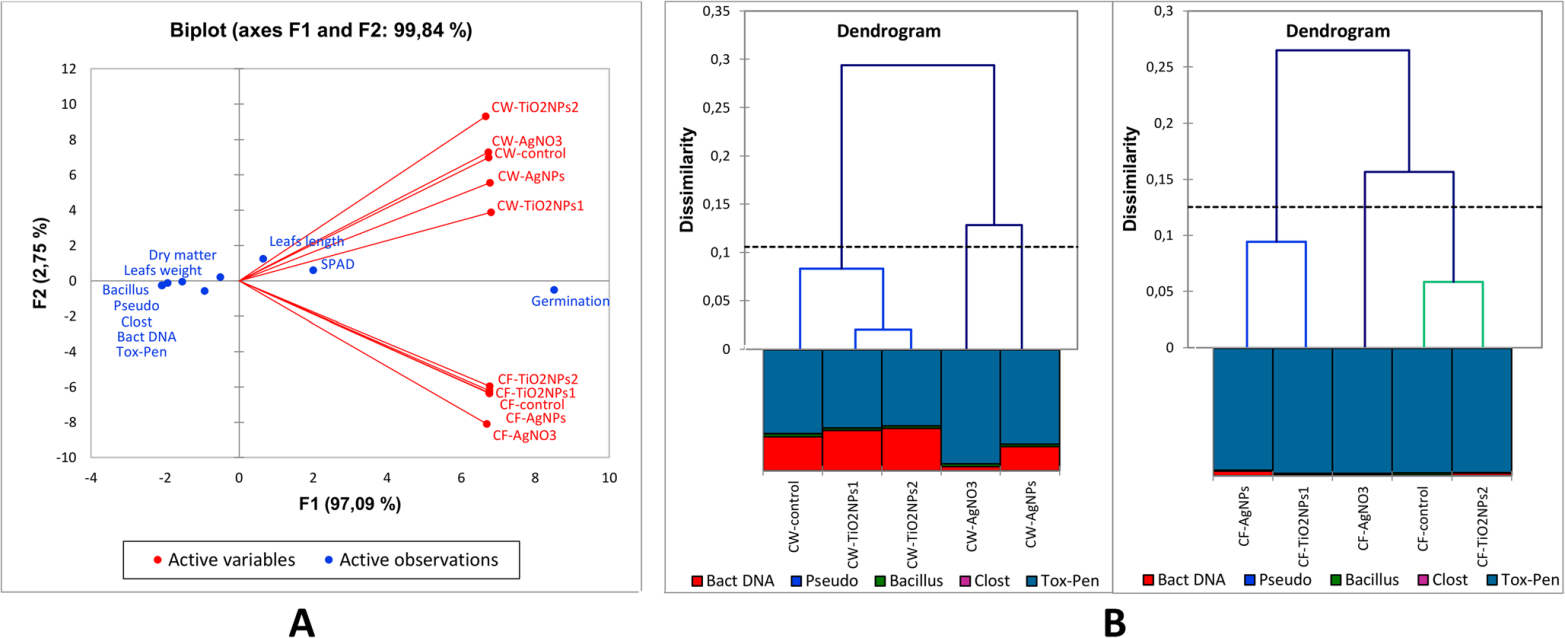
**a** Principal component analysis (PCA) shows correlation between objects (red) and discrimination factors (blue) and **b** agglomerative hierarchical clustering (AHC) describe dissimilarity between objects in microbiological parameters contacts. CW common wheat, CF common flax

The principal component analysis (PCA) presented in Fig. 1a confirmed the lack of correlation in the response to NP between wheat (CW) and flax (CF), but average correlation for CW and strong for CF inside group. The main parameter variable for CW and CF was germination, and in less scale SPAD and leaf biometry. These results indicate that NPs have a strong effect on germination and medium on greenness of leaves irrespective of the Monocot and Dicot form plants. The rhizoplane microbiome parameters were a not strong variable, which indicates a poor microbiome response to NP in soil conditions. This allows us to conclude that in the plant-microbiome system in soil conditions, the more effective response induce NP at the level of plant physiology. Results of agglomerative hierarchical clustering (AHC) presented in Fig. 1b demonstrate differentiation in response of both plant group. In case of wheat, CW-control, CW-TiO_2_NPs1, and CW-TiO_2_NPs2 were not variable, but CW-AgNO_3_ and CW-AgNPs were different. In case of flax, were formed three groups: first CF-control/CF- TiO_2_NPs2, second CF-AgNO_3,_ and third CF-AgNPs/CF-TiO_2_NPs1. Results indicated that AgNO_3_ strong influence on microbiome, nevertheless the plant type was main impact on fate of chemical compounds. Next, grouped in analysis negatively charged AgNPs and TiO_2_NPs1 in CF case indicated important influence of surface charge. However, microbiological AHC method showed some differentiation between those parameters, the PCA indicated smaller weight in these variables with reference to other analysis parameters.

## Conclusions

Our experiment above all shows the importance of the test plants used in evaluation of NP influence. First of all, the response of Monocot and Dicot growth form plants to the tested NPs was different. Secondly, rhizoplane microbiome is dependent on the species of plant, and that the bacteria found in the communities are sensitive to NPs to a varying degree. We also found that the surface charge of metal NPs under soil conditions matter for rhizoplane microbiome. Positively charged NPs significantly decreased the quantity of total bacteria in contrast to negatively charged NPs which increased their quantity in Dicot rhizoplane.
